# Prevalence of Neural Autoantibodies in Paired Serum and Cerebrospinal Fluid in Adult Patients with Drug-Resistant Temporal Lobe Epilepsy of Unknown Etiology

**DOI:** 10.3390/jcm10214843

**Published:** 2021-10-21

**Authors:** Pablo Cabezudo-García, Nicolás L. Ciano-Petersen, Natalia Mena-Vázquez, Jesús Ortega-Pinazo, María J. Postigo-Pozo, Guillermina García-Martín, Helena Antolí-Martínez, Violeta Sánchez-Sánchez, Pablo Quiroga-Subirana, Pedro J. Serrano-Castro, Guillermo Estivill-Torrús

**Affiliations:** 1 Biomedical Research Institute of Málaga-IBIMA, 29010 Málaga, Spain; pablocabezudo@gmail.com (P.C.-G.); lundahl151@gmail.com (N.L.C.-P.); nataliamenavazquez@gmail.com (N.M.-V.); jesusortegapinazo@gmail.com (J.O.-P.); mjpostigopozo@hotmail.com (M.J.P.-P.); guillerminagmartin@gmail.com (G.G.-M.); helenaantoli@gmail.com (H.A.-M.); guillermo.estivill@ibima.eu (G.E.-T.); 2 Neurosciences Clinical Unit, University Regional Hospital of Málaga, 29010 Málaga, Spain; 3 School of Medicine, University of Málaga, 29010 Málaga, Spain; 4 Rheumatology Clinical Unit, University Regional Hospital of Málaga, 29009 Málaga, Spain; 5 Neurology and Neurophysiology Unit, University Hospital Virgen Macarena, 41009 Sevilla, Spain; violetasanchezsanchez@gmail.com; 6 Neurology Unit, Hospitalary Complex Torrecárdenas, 04009 Almería, Spain; pabloquiroga@yahoo.com

**Keywords:** autoimmune epilepsy, autoimmune-associated epilepsy, drug-resistant epilepsy, temporal lobe epilepsy, neural autoantibodies, prevalence

## Abstract

In order to determine the prevalence of neural autoantibodies in adult patients with drug-resistant temporal lobe epilepsy (DRTLE) of unknown etiology, we compared the characteristics of patients with and without autoantibodies and applied antibody predictive scores to the patients. Patients aged ≥18 years with DRTLE of unknown etiology and ≥12 months of evolution were prospectively recruited. Neural autoantibodies in serum and CSF were systematically determined in all patients. We created the ARTE (antibody in drug-resistant temporal lobe epilepsy) score based on the variables associated with the presence of neural autoantibodies. Twenty-seven patients were included. The mean (SD) age in years at the index date was 52 (±14.2) and at epilepsy onset was 32 (±17.1). The mean epilepsy duration was 19 (±12.5) years. Neural autoantibodies were detected in 51.85% (14/27) of patients. The presence of bitemporal, independent, interictal epileptiform discharges (BIIED) had a higher frequency in patients with neural autoantibodies (57.1% vs. 15.4%; *p* = 0.025) as well as those patients with a previous history of status epilepticus (49.2% vs. 0.0%; *p* = 0.007). The ARTE score showed an area under the curve (AUC) of 0.854. Using a cut-off point of ≥1, the sensitivity was 100% and the specificity was 46.1%, whereas when using a cut-off point of ≥3, the results were 35.7% and 100%, respectively. We found a high prevalence of neural autoantibodies in patients with DRTLE of unknown etiology, indicating an autoimmune mechanism. The presence of BIIED and a history of SE in DRTLE of unknown etiology are possible markers for autoimmune-associated epilepsy. The proposed ARTE score requires future validation in larger independent cohorts.

## 1. Introduction

The presence of neural autoantibodies is considered to be one of the hallmarks of autoimmune epilepsy. It was included as a new etiologic category in the ILAE Epilepsy Classification (2017) [1] but is still a matter of debate among experts [2,3].

Several studies with remarkable differences in their methodology attempted to determine the possibility of an autoimmune mechanism in a heterogeneous group of patients with epilepsy of unknown etiology by determining the presence of neural autoantibodies. Many of these studies were performed only in patients with a high suspicion of autoimmune encephalitis [4]. A few studies retrospectively analyzed the available CSF samples with the detection of neural autoantibodies in up to 48.06% of patients [5]. Recently, a systematic review and meta-analysis of the studies, with an a priori low suspicion of autoimmune encephalitis and prospective recruitment, estimated a pooled prevalence of neural autoantibodies in patients with epilepsy of unknown etiology of 7.6% (CI 95%, 4.6–11.2) [6], with a higher prevalence in studies focused on patients with drug-resistant epilepsy. None of the included studies performed a systematic determination of the neural autoantibodies in CSF, leading to the possibility of false negative results [7], and thus a lower prevalence of neural autoantibodies.

A few of these studies tried to determine the epidemiological, clinical, analytical and radiological characteristics of patients harboring neural autoantibodies and to develop predictive scores for the detection of antibodies [8,9,10]. In general, these scores presented a high accuracy but, when applied to a population with different epilepsy characteristics from the original, the sensitivity may have been affected. For example, new-onset epilepsy is predictive for neuronal autoantibodies [8] and is one of the items of the first score to be published, the antibody prevalence in epilepsy (APE) [11], but a recent publication showed that this score lacked sensitivity for patients with a history of epilepsy lasting ≥ 12 months [10]. These studies observed several other frequent variables that conferred a risk for the presence of neural autoantibodies such as drug-resistant epilepsy or a temporal lobe affectation, including the presence of temporal lobe T2 hyperintensities. Patients with these characteristics are common in epilepsy surgery units [12] and the possibility of offering an etiologic treatment with immunotherapy is relevant. 

Considering the above, we conducted a study to determine the prevalence of neural autoantibodies in a group of patients with drug-resistant temporal lobe epilepsy (DRTLE) of unknown etiology and ≥12 months of disease using paired serum and CSF samples as well as comparing the clinical, analytical, electroencephalographic and neuroimaging characteristics between the positive and negative subjects. We also analyzed the testing properties of the previous predictive scores in our patients and proposed a new score for patients with DRTLE.

## 2. Materials and Methods

### 2.1. Design

This was a cross-sectional observational study of a series of patients with DRTLE and at least 12 months of disease. The study was performed in the Epilepsy Unit of the Hospital Regional Universitario de Málaga (EU-HRUM), Spain. The study was approved by the Clinical Research Ethics Committee (CEIC) of the hospital on 23 February 2018 (Code 0672-N-18). All subjects signed an informed consent form.

### 2.2. Patients

Between March 2019 and August 2020, all patients undergoing a follow-up at the EU-HRUM who met the inclusion criteria were consecutively recruited. The inclusion criteria were: a diagnosis of epilepsy according to the ILAE [13]; patients aged ≥18 years; epilepsy onset after 24 months of age; duration of epilepsy of ≥12 months at the index date; temporal lobe epilepsy characterized clinically and by an electroencephalogram (EEG) (at least one EEG with epileptiform discharges in the temporal lobe); brain MRI of ≥1.5 T with an epilepsy protocol [14] without epileptogenic lesions except for hippocampal sclerosis; and drug resistance according to the ILAE definition [15]. Informed consent signatures to perform the lumbar puncture technique, as well as participation in the study, were required. Patients were excluded if they had first-degree relatives with epilepsy, a history of febrile seizures, a history of a moderate to severe head injury before the index date, a history of a neurodevelopmental disorder, a history of infectious meningoencephalitis, an immunosuppressive treatment at the time of sampling, the presence of psychogenic nonepileptic seizures, and findings other than hippocampal sclerosis or nonspecific gliosis in the pathological study of those patients who underwent epilepsy surgery. Patients with hippocampal sclerosis and epilepsy onset before 20 years of age were also excluded [16].

### 2.3. Protocol

All patients underwent anamnesis and a physical examination on the inclusion date, which was also the index date. On the index date, blood was extracted and CSF was obtained by a lumbar puncture. The blood collection included plasma and serum. The samples were stored at 4 °C and immediately sent to the laboratory for processing.

### 2.4. Prevalence of Neural Autoantibodies

The main outcome was to determine the number of patients (%) with a positive result for neural autoantibodies related to autoimmune epilepsy [17] in serum or CSF. The antibody profile of each patient with a positive result was registered. The antibody profile corresponded with the medium where it was detected and the type of antibody.

Neural autoantibodies were determined by IIFT on transfected cells as a cell-based assay (CBA) using BIOCHIP Mosaics (IIFT Autoimmune Encephalitis Mosaic6 EUROIMMUN) and the detection of antineuronal antibodies was achieved against the following surface antigens: contactin-associated protein-like 2 (CASPR2), dipeptidyl-peptidase-like protein-6 (DPPX), leucine-rich glioma-inactivated 1 (LGI1), N-methyl-D-aspartate receptors (NMDAR1/R2), anti-γ-aminobutyric acid B receptor (GABABR), and α-amino-3-hydroxy-5-methyl-4-isoxazole propionic acid receptor (AMPAR). For the determination of the antibodies directed against the intracellular antigens, immunoblotting (EUROLINE test kit, Paraneoplastic Neurologic Syndromes 12 Ag, EUROIMMUN) was performed, which included amphiphysin, recoverin, titin, Zic4, SOX1, CV2, paraneoplastic ma antigen 2 (PNMA2, Ma2/Ta), Ri, Hu, Yo, the 65 kD isoform of glutamic acid decarboxylase (GAD65) and Tr (DNER). The results were analyzed using EuroLineScan (EUROIMMUN) and subsequently confirmed by IIFT on tissue or CBA (EUROIMMUN, Lübeck, Germany). Of all the above antibodies, only those related to autoimmune epilepsy [17] were considered to be a positive result. The detection of GAD65 either by immunoblotting or IIFT was considered significant as they corresponded with high titers when quantitative methods were used [18]. All determinations were performed in both serum and CSF.

### 2.5. Baseline Characteristics

At the index date, the neurologist collected the epidemiological, clinical, electroencephalographic, analytical and neuroimaging data (the baseline characteristics). The collected variables, several of which were based on previous studies [8] were the following: sex; age; age at epilepsy onset; duration of epilepsy in years; the presence of systemic cancer (diagnosed within 5 years of neurological symptom onset); a history of aseptic-lymphocytic meningoencephalitis prior to or at the onset of epilepsy; a personal history of autoimmune diseases (APLAb, lupus, DM type 1, myasthenia gravis, Sjogren’s, RA, Crohn’s, celiac, UC, Hashimoto’s, Graves, and psoriasis); neuropsychiatric changes (agitation, aggressiveness, and emotional lability); cognitive symptoms; dysautonomia (sustained atrial tachycardia or bradycardia); orthostatic hypotension (≥20 mmHg decrease in systolic pressure or ≥10 mmHg decrease in diastolic pressure within 3 min of quiet standing); hyperhidrosis; persistently labile blood pressure; ventricular tachycardia; cardiac asystole or gastrointestinal dysmotility after epilepsy onset, and not attributable to other causes; focal neurological findings such as speech problems; a history of epilepsy surgery; a history of status epilepticus [19]; the number of currently antiepileptic drugs; focal seizures with and without impaired consciousness; generalized tonic–clonic seizures; autonomic seizures; bitemporal, independent, interictal epileptiform discharges (BIIED); video-electroencephalography (vEEG) recording; CSF inflammation (elevated CSF protein of >50 mg/dL and/or lymphocytic pleocytosis of >5 cells/microL if the total number of CSF RBC was <1000 cells/microL); specific oligoclonal bands; findings suggestive of encephalitis from MRI (T2/FLAIR hyperintensity restricted to one or both medial temporal lobes or multifocal in grey matter, white matter, or both compatible with demyelination or inflammation) and hippocampal sclerosis. The presence of hypo/hypermetabolism in patients with an available brain PET was also registered.

### 2.6. Antibody Prevalence Scores

The area under the curve (AUC) and testing properties for the antibody in the drug-resistant temporal lobe epilepsy (ARTE) score were calculated. The proposed ARTE score was constructed based on the variables associated with the presence of neural autoantibodies in our study and other scores. We also probed the utility of published antibody prevalence scores in our patients (using the recommended cut-off points): Antibody prevalence in epilepsy and encephalopathy (APE2) score [8], antibody prevalence in epilepsy before surgery (APES) score [9], and antibodies contributing to focal epilepsy signs and symptoms (ACES) score [10]. The punctuation of these scores was calculated for each patient.

### 2.7. Statistical Analysis

A descriptive analysis of neural autoantibodies regarding the presence, type and medium detected was conducted as well as for the epidemiological and clinical characteristics of the included patients. The qualitative variables were expressed as an absolute number and percentage and quantitative variables as the mean and standard deviation (SD) or as the median and interquartile range (IQR), according to the normality adjustment using the Kolmogorov–Smirnov test. Χ2 and a Student’s *t*-test or a Mann–Whitney *t*-test were performed to compare the main characteristics between the patients with and without autoantibodies. We constructed a receiver operating characteristic (ROC) curve for the different antibody prevalence scores. For all analyses, a *p*-value of <0.05 was considered to indicate significance and the statistical program R2.4-0 was used.

## 3. Results

### 3.1. Baseline Characteristics and the Frequency of Neural Autoantibodies

The study population comprised 27 patients with DRTLE. The majority of the participants were women (63%) with a mean (SD) age at the index date of 52 (±14.2) years. The mean (SD) age of epilepsy onset was 32 (±17.1) years and the mean epilepsy duration was 19 (±12.5) years. A total of 14 (51.85%) patients presented a positive result (Table 1). Both groups were balanced in terms of epidemiological, clinical, analytical, and neuroimaging characteristics. However, the presence of BIIED had a higher frequency in antibody-positive patients (57.1% vs. 15.4%; *p* = 0.025) as well as the antecedent of SE (49.2% vs. 0.0%; *p* = 0.007). Regarding SE, two of the patients (patients 2 and 5) presented with a new-onset convulsive SE that required mechanical ventilation in the intensive care unit (ICU), two presented with recurrent aphasic status epilepticus (patients 4 and 13), one presented with recurrent “ambulatory” nonconvulsive SE (patient 11) and one presented with a unique episode of convulsive SE (patient 7) after the epilepsy diagnosis that did not require the ICU. No patient presented with temporal lobe T2 hyperintensities or systemic cancer and only one patient presented with dysautonomia in the form of orthostatic hypotension (patient 9). All the autonomic seizures were focal autonomic seizures with an epigastric sensation. As only six patients (five with neural autoantibodies) had a brain PET, this variable was not included in the statistical comparison. All patients presented temporal lobe hypometabolism.

### 3.2. Neural Autoantibodies Profile

The autoantibodies (Table 2) were detected with a higher frequency in serum (12/14, 85.71%) than in CSF (4/14, 28.57%). Only two (14%) patients had a positive result exclusively in CSF (both DPPX). Two (14%) patients presented positivity for ≥ 2 antibodies in serum. The most frequent autoantibody found was serum NMDAR in 4/14 (35.71%) followed by serum LGI1 in 3/14 (21.42%) (see Supplementary Figures S1 and S2 for representative images).

### 3.3. Sensitivity and Specificity of Antibody Predictive Scores

For each patient we calculated the APE2, APES, ACES and ARTE scores (Table 2). In our sample, the areas under the curve (AUC) for the APE2, APES and ACES scores were 0.640 (95% CI, 0.430–0.851; *p* = 0.216), 0.736 (95% CI, 0.545–0.928; *p* = 0.037) and 0.758 (95% CI, 0.576–0.940; *p* = 0.023), respectively. The application of the APE2 score (cut-off point of ≥4) had a sensitivity of 21.4% (95% CI, 5.7–51.1) and a specificity of 100% (95% CI, 71.6–99.2), whereas the sensitivities and specificities for the APES score (cut-off point of ≥4) were 42.86% (CI 95%, 18.66–70.3) and 84.6% (CI 95%, 53.6–97.2), respectively. We determined the sensitivity and specificity for the ACES score using a cut-off point of ≥2 with results of 64.2% (CI 95%, 35.6–86.0) and 76.9% (CI 95% 45.9–93.8), respectively. Using the ACES score with a cut-off point of ≥1, the sensitivity was 92.86% and the specificity was 30.77%.

**Figure 1:**
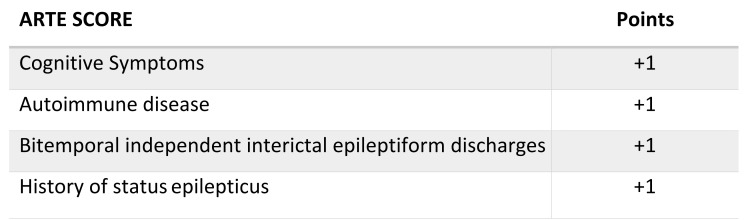
ARTE score.

The proposed score, ARTE (Figure 1), was constructed based on the variables associated with the neural autoantibodies in our study (the presence of BIIED and a history of SE). The remaining variables were selected after testing the different models, aiming to obtain the best AUC and clinical utility. The presence of each scored one point to a maximum score of four. The calculated area under the curve (AUC) in the ARTE score was 0.854 (CI 95%, 0.716–0.993; *p* = 0.002). The testing properties of the ARTE score using different cut-off points are presented in Table 3.

## 4. Discussion

In our study, the prevalence of neural autoantibodies in patients with DRTLE of unknown etiology was high compared with previous results [6] with more than half the subjects presenting a positive result. This confirmed our initial hypothesis that patients with these characteristics are at risk of the presence of neural autoantibodies because drug resistance is a hallmark of autoimmune seizures [20] and there is a predilection of autoimmune encephalitis affecting the temporal lobe and limbic system structures [7,21]. Conducting the systematic determination of the antibodies in CSF also entailed a mild increase in the proportion of positive patients in our study. The most frequent antibody found in our patients was anti-NMDAR, but this was only found in serum. It should be noted that the presence of the anti-NDMAR(GluN1) IgG subclass in CSF is required for the diagnosis of definite anti-NMDAR encephalitis and only positive results in serum can be considered to be unspecified or as false positives [7]. Thus, the role in the epilepsy of autoantibodies detected only in serum, especially in patients without overt encephalitis (autoimmune-associated epilepsy), should be addressed in future studies [22].

Regarding the differences between patients with and without autoantibodies, we found a higher frequency of BIIED in the former without differences in the number of patients with long-term vEEG or the duration of epilepsy. A similar finding was also observed in the work of Casciato et al. [23] where the majority of patients with limbic encephalitis were radiological with EEG involvement in both temporal lobes, highlighting a bitemporal involvement in the immune-mediated processes. A history of SE was also observed with a higher frequency in positive patients who suffered from NORSE or recurrent focal SE. The literature has shown in different studies that SE, including NORSE, is not an uncommon manifestation of autoimmune encephalitis [24,25]. The studies regarding the prognostic factors in SE showed that recurrent SE had a frequency of up to 37% and that a remote symptomatic etiology was a risk factor [26]. A chronic and untreated autoimmune process could be taken to be a remote symptomatic cause, explaining the recurrent focal SE in our patients. Although the principal hypothesis is that antibodies are causative of SE, it would not be imprudent to think that the presence of neural autoantibodies is a consequence of an antigenic exposure in the context of neuronal injury. For example, patients with herpes simplex encephalitis frequently develop anti-NDMAR and other neural autoantibodies in CSF in direct relation to the degree of neuronal damage [27], and there is evidence of anti-NMDAR autoantibodies targeted to subunits other than GluN1-ATD in the serum of patients who had suffered a stroke [28]. It would be interesting to carry out a study on the detection of neuronal autoantibodies in status epilepticus other than the suspected autoimmune. We did not find any differences in the other characteristics between the positive and negative patients, which may partly be due to the low number of patients included in the study.

We applied several published predictive scores using the recommended cut-off points for the detection of neural autoantibodies in our sample. The APE2 score [8] showed a specificity of 100% but a low sensitivity, similar to that applied to patients with no new-onset epilepsy in Bruijn et al. [10]. Likewise, a scale that showed sensitivity for patients with an epilepsy duration of at least 12 months, the ACES score [10], did not show a high sensitivity in our group. This most likely occurred because the presence of variables such as temporal lobe T2 hyperintensities or autonomic symptoms used to calculate this score were under-represented in our patients. MRI is often unremarkable, even in overt autoimmune encephalitis, and, usually, temporal lobe hyperintensities are only present in the acute phase of the disease [21]. We only included patients with chronic epilepsy in our study with a mean duration of epilepsy of 19 years. Due to this, in most of the included patients, the MRI at the epilepsy onset was not performed or was not available for revision. It may have been possible to detect the temporal hyperintensities in a few cases if they had been available. The APES score [9], a modified score from APE2 tailored to patients with drug-resistant epilepsy, also presented a low sensitivity in our patients. This could be explained, at least partially, by the fact that several of the variables used in this score were not registered in our study (for example, “systemic”) and that only a few patients in our study (20%) had the available cerebral PET results. We proposed a new score for chronic DRTLE of an unknown etiology, the ARTE score. A cut-off point of ≥1 showed a 100% sensitivity but a low specificity, whereas using a cut-off point of ≥3 showed a high specificity but a low sensitivity. This high sensitivity and specificity achieved in our population was presumably due to the fact that the score was constructed based on this population and with a small number of patients. There is a likelihood that this would not be the case if applied to other independent cohorts, as demonstrated with previously developed scores in cohorts with a high number of patients. The ARTE score must be validated in an independent cohort of patients, ideally with similar characteristics and where CSF is systematically analyzed. Nonetheless, we think that the AUC and testing properties obtained in our sample are interesting.

Our work has several limitations. First, our study included only a small number of patients. This was mainly due to the methodology of our work. We selected patients with concrete epilepsy characteristics and performed a systematic and prospective CSF sampling (in previous published studies with a higher number of patients, a CSF assessment was only usually performed when there is a positive result in serum or when CSF is obtained on a clinical basis from patients with suspected autoimmune encephalitis). The points in our methodology were recently reinforced by members of the ILAE Autoimmune group in a recent review [22]. An adequate strategy to increase the number of patients would be to perform a multicenter study and that is the next step in our group, supported by the results presented in this paper. Second, the ARTE score requires validation from an independent cohort to obtain a reliable conclusion; nonetheless, a good AUC was obtained in our patients. Finally, another limitation was the use of commercial kits for the detection of autoantibodies with the limitations of the false positives and negatives that these entail. To reduce these possibilities, several samples were sent to a reference laboratory for confirmation. We also could not perform the determination of a few antibodies, such as anti-GlyR, as commercial kits were not available.

## 5. Conclusions

There is a high prevalence of neural autoantibodies in serum and CSF in patients with DRTLE of unknown etiology and more than 12 months of disease. The presence of BIIED and the history of status epilepticus could be a marker of autoimmune-associated epilepsy. These results and the proposed ARTE score need future prospective validation in studies with a greater number of patients.

## Figures and Tables

**Table 1:** Baseline characteristics of positive and negative DRTLE patients *.

Variables	Anti-Neural Positive *n* = 14	Anti-Neural Negative *n* = 13	*p*-Value
Female sex, n (%)	9 (64.3%)	8 (61.5%)	0.883
Epilepsy onset age in years, mean (SD)	34.1 (17.1)	31.2 (17.7)	0.672
Age in years, mean (SD)	51.5 (15.3)	52.7 (13.5)	0.829
Epilepsy duration years, mean (SD)	17.2 (11.2)	21.3 (14.0)	0.410
Aseptic meningoencephalitis, n (%)	1 (7.1%)	0 (0.0%)	0.326
Autoimmune disease, n (%)	6 (42.9%)	2 (15.4%)	0.118
Neuropsychiatric changes, n (%)	7 (50.0%)	4 (30.8%)	0.310
Cognitive symptoms, n (%)	9 (64.3%)	5 (38.5%)	0.180
Dysautonomia, n (%)	1 (7.1%)	0 (0.0%)	0.326
Speech problems, n (%)	2 (14.3%)	1 (7.7%)	0.586
Epilepsy Surgery, n (%)	1 (7.1%)	1 (7.7%)	0.957
Status epilepticus, n (%)	6 (42.9%)	0 (0.0%)	0.007
Number of AEDs, mean (SD)	2.4 (1.0)	2.4 (0.9)	0.932
Focal seizures without IA, n (%)	8 (57.1%)	5 (38.5%)	0.332
Focal seizures with IA, n (%)	12 (85.7%)	13 (100%)	0.157
GTC seizures, n (%)	11 (78.6%)	8 (68.5%)	0.333
Autonomic seizures, n (%)	5 (35.71%)	3 (23.1%)	0.472
Bitemporal independent interictal discharges, n (%)	8 (57.1%)	2 (15.4%)	0.025
Performed vEEG, n (%)	6 (42.9%)	7 (53.8%)	0.568
CSF inflammation, n (%)	4 (28.6%)	1 (7.7%)	0.163
Specific OGB, n (%)	1 (7.7%)	1 (8.3%)	0.953
Hippocampal sclerosis, n (%)	0 (0.0%)	2 (15.4%)	0.127

Abbreviations: DRTLE, drug-resistant temporal lobe epilepsy; SD, standard deviation; AED, antiepileptic drugs; IA, impaired awareness; GTC, generalized tonic-clonic; vEEG, video-electroencephalography; CSF, cerebrospinal fluid; OGB, oligoclonal bands. * Only characteristics present in at least one patient are represented.

**Table 2:** Neural autoantibodies profile and antibody prevalence score results.

Positive Patients	Serum	CSF	APE2 Score	APES Score	ACES Score	ARTE Score
Patient 1	GAD65 + NMDAR	-	2	4	1	1
Patient 2	LGI1	-	3	5	2	2
Patient 3	AMPAR	AMPAR	2	3	1	1
Patient 4	CV2	-	2	4	1	3
Patient 5	LGI1	-	2	3	1	3
Patient 6	GAD65	-	4	3	2	1
Patient 7	NMDAR	-	3	3	2	3
Patient 8	GABA_B_R	-	3	2	3	2
Patient 9	NMDAR	-	4	4	3	2
Patient 10	-	DPPX	2	3	2	3
Patient 11	-	DPPX	4	4	3	4
Patient 12	AMPAR	AMPAR	3	3	0	1
Patient 13	NMDAR	-	3	3	2	2
Patient 14	LGI1 + DPPX + GABA_B_R		3	2	2	1

Abbreviations: CSF, cerebrospinal fluid; APE2, antibody prevalence in epilepsy and encephalopathy; APES, antibody prevalence in epilepsy before surgery; ACES, antibodies contributing to focal epilepsy signs and symptoms; ARTE, antibodies in drug-resistant temporal lobe epilepsy; GAD65: 65 kD isoform of glutamic acid decarboxylase; NMDAR: N-methyl-D-aspartate receptor; LGI1: leucine-rich glioma-inactivated 1; AMPAR:α-amino-3-hydroxy-5-methyl-4-isoxazole propionic acid receptor; GABA_B_R: anti-γ-aminobutyric acid B receptor; DPPX: dipeptidyl-peptidase-like protein-6.

**Table 3:** Testing Properties of the ARTE Score Using Different Cutoff Values.

Cutoff	N (%)	Sensitivity (95% CI)	Specificity (95% CI)	PPV (95% CI)	NPV (95% CI)
≥1 points	21 (77.7)	100% (76.8–100)	46.1% (19.2–74.8)	66.6% (54.7–76.7)	100%
≥2 points	11 (40.7)	64.2% (35.1–87.2)	84.6% (54.5–98.0)	81.8% (54.2–94.4)	68.7% (51.2–82.1)
≥3 points	5 (18.5)	35.7% (12.7–64.8)	100% (75.2–100)	100%	66.6% (46.0–83.4)

## Data Availability

The data used to support the findings of this study are included within the article and the Supplementary Material.

## Supplementary Materials

The following are available online at https://www.mdpi.com/article/10.3390/jcm10214843/s1, Figure S1:Representative images from BIOCHIP Mosaic analysis using *the IIFT Autoimmune Encephalitis Mosaic 6 kit* (EUROIMMUN, Lübeck, Germany); Figure S2:The image corresponds to serum samples from patient 6 (GAD65 +) and patient 4 (CV2 +).

## References

[1] Scheffer I.E., Berkovic S., Capovilla G., Connolly M.B., French J., Guilhoto L., Hirsch E., Jain S., Mathern G.W., Moshé S.L. (2017). ILAE classification of the epilepsies: Position paper of the ILAE Commission for Classification and Terminology. Epilepsia.

[2] Geis C., Planagumà J., Carreño M., Graus F., Dalmau J. (2019). Autoimmune seizures and epilepsy. J. Clin. Investig..

[3] Steriade C., Britton J., Dale R.C., Gadoth A., Irani S.R., Linnoila J., McKeon A., Shao X.-Q., Venegas V., Bien C.G. (2020). Acute symptomatic seizures secondary to autoimmune encephalitis and autoimmune-associated epilepsy: Conceptual definitions. Epilepsia.

[4] Kuehn J.C., Meschede C., Helmstaedter C., Surges R., von Wrede R., Hattingen E., Vatter H., Elger C.E., Schoch S., Becker A.J. (2020). Adult-onset temporal lobe epilepsy suspicious for autoimmune pathogenesis: Autoantibody prevalence and clinical correlates. PLoS ONE.

[5] Zhang W., Bu H., Li Y., Han X., He J., Jia L., Wang W. (2020). Development and validation of a predictive model for the diagnosis of neural antibody-mediated epilepsy/ seizure in patients with new-onset seizure or established epilepsy. Seizure.

[6] Cabezudo-García P., Mena-Vázquez N., Ciano-Petersen N.L., García-Martín G., Estivill-Torrús G., Serrano-Castro P.J. (2021). Prevalence of neural autoantibodies in epilepsy of unknown etiology: Systematic review and meta-analysis. Brain Sci..

[7] Graus F., Titulaer M.J., Balu R., Benseler S., Bien C.G., Cellucci T., Cortese I., Dale R.C., Gelfand J.M., Geschwind M. (2016). A clinical approach to diagnosis of autoimmune encephalitis. Lancet Neurol..

[8] Dubey D., Pittock S.J., McKeon A. (2019). Antibody Prevalence in Epilepsy and Encephalopathy score: Increased specificity and applicability. Epilepsia.

[9] Li Y., Tymchuk S., Barry J., Muppidi S., Le S. (2021). Antibody Prevalence in Epilepsy before Surgery (APES) in drug-resistant focal epilepsy. Epilepsia.

[10] de Bruijn M.A.A.M., Bastiaansen A.E.M., Mojzisova H., van Sonderen A., Thijs R.D., Majoie M.J.M., Rouhl R.P.W., van Coevorden-Hameete M.H., de Vries J.M., Lopetegi A.M. (2021). Antibodies Contributing to Focal Epilepsy Signs and Symptoms Score. Ann. Neurol..

[11] Dubey D., Alqallaf A., Hays R., Freeman M., Chen K., Ding K., Agostini M., Vernino S. (2017). Neurological autoantibody prevalence in epilepsy of unknown etiology. JAMA Neurol..

[12] Sultana B., Panzini M.A., Veilleux Carpentier A., Comtois J., Rioux B., Gore G., Bauer P.R., Kwon C.-S., Jetté N., Josephson C.B. (2021). Incidence and Prevalence of Drug-Resistant Epilepsy: A Systematic Review and Meta-analysis. Neurology.

[13] Fisher R.S., Acevedo C., Arzimanoglou A., Bogacz A., Cross J.H., Elger C.E., Engel J., Forsgren L., French J.A., Glynn M. (2014). ILAE Official Report: A practical clinical definition of epilepsy. Epilepsia.

[14] Bernasconi A., Cendes F., Theodore W.H., Gill R.S., Koepp M.J., Hogan R.E., Jackson G.D., Federico P., Labate A., Vaudano A.E. (2019). Recommendations for the use of structural magnetic resonance imaging in the care of patients with epilepsy: A consensus report from the International League Against Epilepsy Neuroimaging Task Force. Epilepsia.

[15] Kwan P., Arzimanoglou A., Berg A.T., Brodie M.J., Hauser W.A., Mathern G., Moshé S.L., Perucca E., Wiebe S., French J. (2010). Definition of drug resistant epilepsy: Consensus proposal by the ad hoc Task Force of the ILAE Commission on Therapeutic Strategies. Epilepsia.

[16] Bien C.G., Elger C.E. (2007). Limbic encephalitis: A cause of temporal lobe epilepsy with onset in adult life. Epilepsy Behav..

[17] Husari K.S., Dubey D. (2019). Autoimmune Epilepsy. Neurotherapeutics.

[18] Graus F., Saiz A., Dalmau J. (2020). GAD antibodies in neurological disorders—Insights and challenges. Nat. Rev. Neurol..

[19] Trinka E., Cock H., Hesdorffer D., Rossetti A.O., Scheffer I.E., Shinnar S., Shorvon S., Lowenstein D.H. (2015). A definition and classification of status epilepticus—Report of the ILAE Task Force on Classification of Status Epilepticus. Epilepsia.

[20] Cabezudo-García P., Mena-Vázquez N., Villagrán-García M., Serrano-Castro P.J. (2018). Efficacy of antiepileptic drugs in autoimmune epilepsy: A systematic review. Seizure.

[21] Kelley B.P., Patel S.C., Marin H.L., Corrigan J.J., Mitsias P.D., Griffith B. (2017). Autoimmune encephalitis: Pathophysiology and imaging review of an overlooked diagnosis. Am. J. Neuroradiol..

[22] Steriade C., Gillinder L., Rickett K., Hartel G., Higdon L., Britton J., French J. (2021). Discerning the Role of Autoimmunity and Autoantibodies in Epilepsy: A Review. JAMA Neurol..

[23] Casciato S., Morano A., Fattouch J., Fanella M., Avorio F., Albini M., Basili L.M., Irelli E.C., Viganò A., Risi M.D. (2019). Factors underlying the development of chronic temporal lobe epilepsy in autoimmune encephalitis. J. Neurol. Sci..

[24] De Bruijn M.A.A.M., Van Sonderen A., Van Coevorden-Hameete M.H., Bastiaansen A.E.M., Schreurs M.W.J., Rouhl R.P.W., van Donselaar C.A., Majoie M.H.J.M., Neuteboom R.F., Sillevis Smitt P.A.E. (2019). Evaluation of seizure treatment in anti-LGI1, anti-NMDAR, and anti-GABABR encephalitis. Neurology.

[25] Gaspard N., Foreman B.P., Alvarez V., Cabrera Kang C., Probasco J.C., Jongeling A.C., Meyers E., Espinera A., Haas K.F., Schmitt S.E. (2015). New-onset refractory status epilepticus: Etiology, clinical features, and outcome. Neurology.

[26] Sculier C., Gaínza-Lein M., Sánchez Fernández I., Loddenkemper T. (2018). Long-term outcomes of status epilepticus: A critical assessment. Epilepsia.

[27] Armangue T., Spatola M., Vlagea A., Mattozzi S., Cárceles-Cordon M., Martinez-Heras E., Llufriu S., Muchart J., Erro M.E., Abraira L. (2018). Spanish Herpes Simplex Encephalitis Study Group. Frequency, symptoms, risk factors, and outcomes of autoimmune encephalitis after herpes simplex encephalitis: A prospective observational study and retrospective analysis. Lancet Neurol..

[28] Javidi E., Magnus T. (2019). Autoimmunity After Ischemic Stroke and Brain Injury. Front. Immunol..

