# Supplementary material for: Prevalence of Neural Autoantibodies in Paired Serum and Cerebrospinal Fluid in Adult Patients with Drug-Resistant Temporal Lobe Epilepsy of Unknown Etiology
Source: J Clin Med. 2021 Oct 21;10(21):4843. doi: 10.3390/jcm10214843 (PMC8584597; doi:10.3390/jcm10214843)
Supplement: Supplementary file 1 [file jcm-10-04843-s001.zip › jcm-1416453-supplementary.pdf]

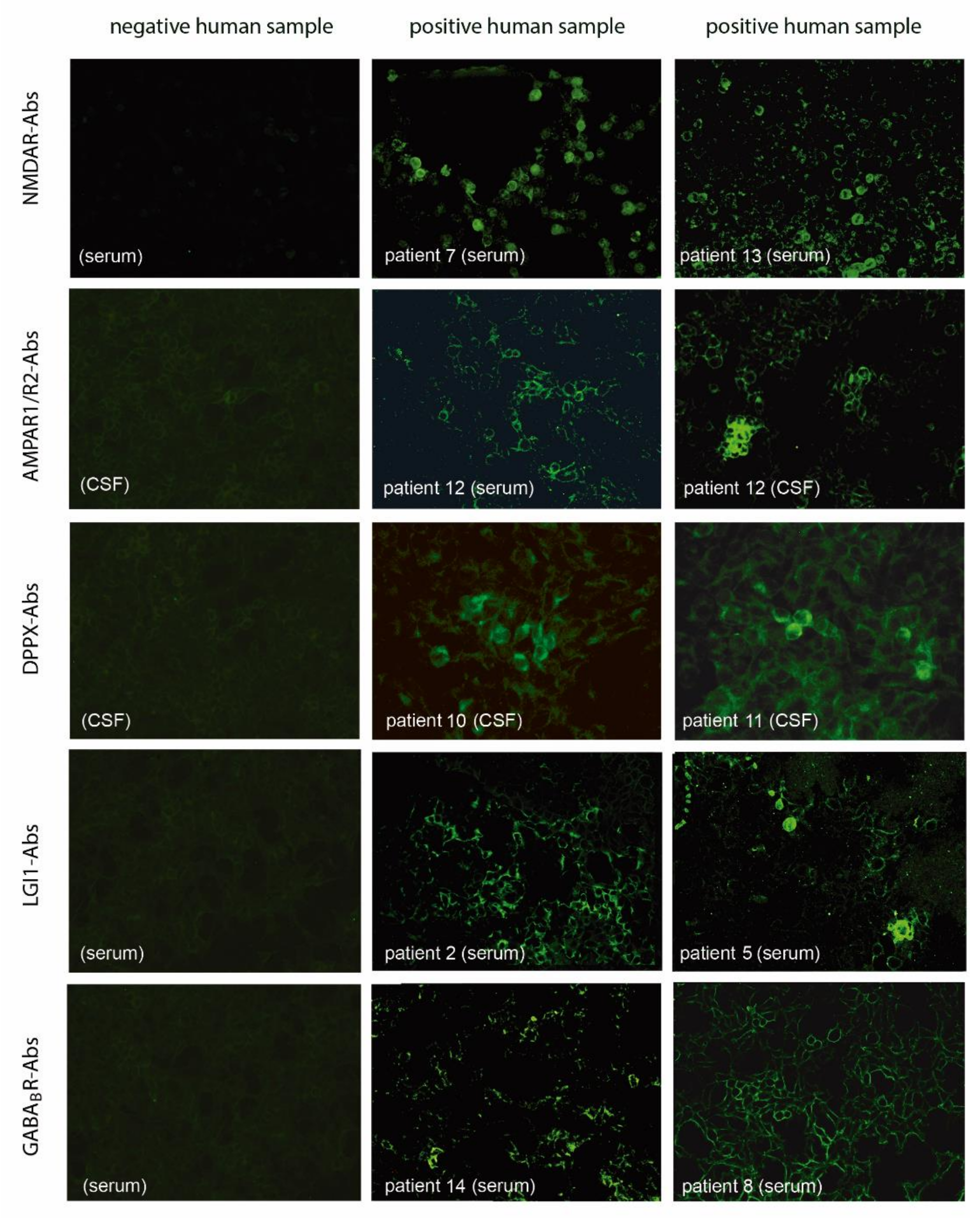

Figure S1. Representative images from BIOCHIP Mosaic analysis using *the IIFT Autoimmune Encephalitis Mosaic 6 kit* (EUROIMMUN, Lübeck, Germany). Serum and CSF were processed according to the manufacturer's instructions. The patient number is indicated in each representative serum or CSF positive sample, as cited in the main text. Negative human samples correspond to serum or CSF samples with no detected immunofluorescence of corresponding antibodies. Images were captured with an Olympus BX51 microscope equipped with a DP70 digital camera (Olympus Europa SE & Co. KG, Hamburg, Germany).

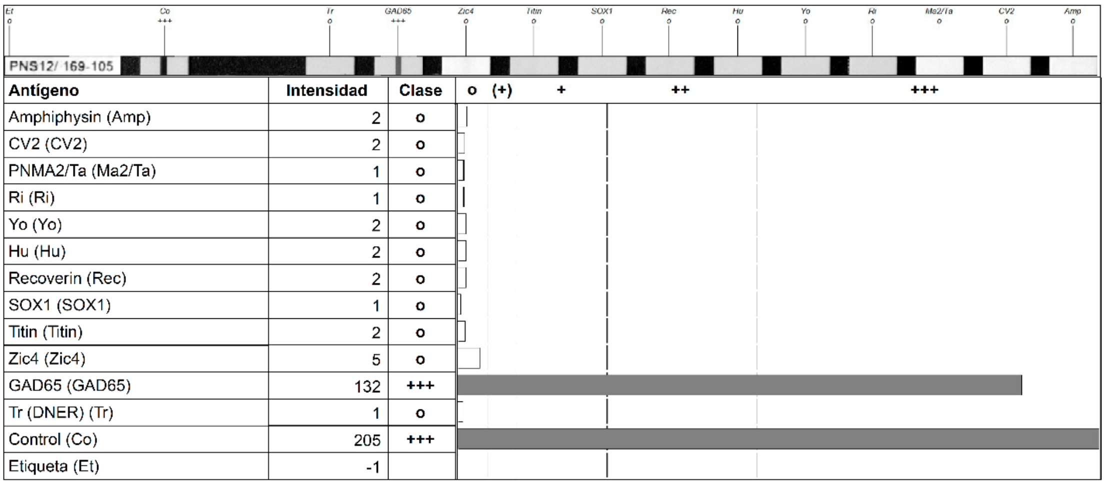

Antígeno	Intensidad	Clase
Amphiphysin (Amp)	2	o
CV2 (CV2)	2	o
PNMA2/Ta (Ma2/Ta)	1	o
Ri (Ri)	1	o
Yo (Yo)	2	o
Hu (Hu)	2	o
Recoverin (Rec)	2	o
SOX1 (SOX1)	1	o
Titin (Titin)	2	o
Zic4 (Zic4)	5	o
GAD65 (GAD65)	132	+++
Tr (DNER) (Tr)	1	o
Control (Co)	205	+++
Etiqueta (Et)	-1	

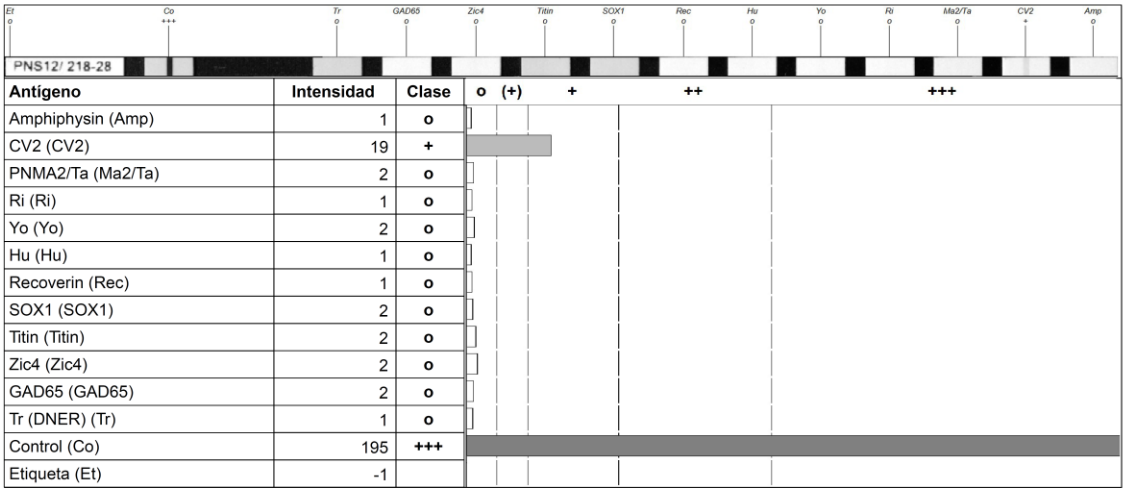

Antígeno	Intensidad	Clase
Amphiphysin (Amp)	1	o
CV2 (CV2)	19	+
PNMA2/Ta (Ma2/Ta)	2	o
Ri (Ri)	1	o
Yo (Yo)	2	o
Hu (Hu)	1	o
Recoverin (Rec)	1	o
SOX1 (SOX1)	2	o
Titin (Titin)	2	o
Zic4 (Zic4)	2	o
GAD65 (GAD65)	2	o
Tr (DNER) (Tr)	1	o
Control (Co)	195	+++
Etiqueta (Et)	-1	

Figure S2. Representative immunoblot strips and immunodetection using the *EUROLINE test kit*, *Paraneoplastic Neurologic Syndromes 12Ag*, and *EuroLineScan* system (EUROIMMUN, Lübeck, Germany). The image corresponds to serum samples from patient 6 (GAD65 +) and patient 4 (CV2 +).
